# Differentiation of *stx1A* gene for detection of *Escherichia coli* serotype O157: H7 and *Shigella dysenteriae* type 1 in food samples using high resolution melting curve analysis

**DOI:** 10.1002/fsn3.1649

**Published:** 2020-05-28

**Authors:** Babak Pakbin, Afshin Akhondzadeh Basti, Ali Khanjari, Leila Azimi, Abdollah Karimi

**Affiliations:** ^1^ Department of Food Hygiene and Quality of Control Faculty of Veterinary Medicine University of Tehran Tehran Iran; ^2^ Pediatric Infections Research Center Research Institute of children’s Health Shahid Beheshti University of Medical Sciences Tehran Iran

**Keywords:** *Escherichia coli* O157: H7, food sample, HRMA, *Shigella dysenteriae* type 1, *stx1A* gene

## Abstract

*Escherichia coli* serotype O157: H7 and *Shigella dysenteriae* type 1 as the Shiga toxin‐producing bacteria cause some acute gastrointestinal and extraintestinal diseases such as hemorrhagic uremic syndrome and bloody diarrhea in human. *Stx* genes are the key virulence factors in these pathogens. The aim of this study was to develop HRMA assay to differentiate *stx1A* gene for detection of *E. coli* serotype O157: H7 and *Sh. dysenteriae* type 1 and determine the prevalence of these pathogens in food samples using this method. PCR‐HRMA assay and gold standard methods have been carried out for identification of pathogens among 135 different food samples. We found HRMA method a sensitive and specific assay (100 and 100%, respectively) for differentiation of *stx1A* gene, consequently, detection of these pathogens in food samples. Also, the highest prevalence of *E. coli* serotype O157: H7 and *Sh. dysenteriae* type 1 harboring *stx1A* gene was observed in raw milk and vegetable salad samples, respectively. HRMA as a rapid, inexpensive, sensitive and specific method is suggested to be used for differentiation of *stx1A* gene to detect *E. coli* serotype O157: H7 and *Sh. dysenteriae* type 1 as the key pathogens for safety evaluation of food samples.

## Graphical abstract

In this paper, we developed high resolution melting curve analysis method to differentiate *stx1A* gene to detect *Escherichia coli* serotype O157: H7 and *Shigella dysenteriae* strains in food samples. We found this method specific and sensitive for detection of these pathogens in food samples. Also, we investigated the prevalence of these foodborne pathogens in food samples using this method. We observed the highest prevalence of *E. coli* serotype O157: H7 and *Sh. dysenteriae* type 1 in raw milk and vegetable salad samples, respectively. We found this method appropriate for detection of these pathogens in naturally contaminated food samples.

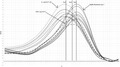

## 1 Introduction

Foodborne pathogens cause annually many illnesses, hospitalizations, and deaths worldwide. Some of these pathogens leading to intra and extraintestinal infections in human such as *Escherichia coli* O157: H7 and *Shigella dysenteriae* type 1 causing hemorrhagic uremic syndrome (HUS) and bloody diarrhea are transmitted by food and drinks (Söderqvist, Lambertz, Vågsholm, & Boqvist, 2016). *E. coli* and *Sh. dysenteriae* are gram‐negative, nonspore forming and rod‐shaping bacteria belonging to Enterobacteriaceae family. Some serotypes of these bacteria recently recognized as the prominent threatening foodborne pathogens (Dallman et al., 2015). Shiga toxin‐producing bacteria include these pathogens and some other serotypes of *E. coli* releasing Shiga toxin proteins. Shiga toxin is known as one of the most potent bacterial toxins encoded by *stx* gene group (Adams et al., 2016). This toxin consists of two subunits including A and B with injuring ribosome (inhibition of protein synthesis) and binding to the cellular receptor functionalities, respectively. B subunit of this toxin binds to the GB_3_ receptor located on the kidney endothelial cells leading to renal failure (Pezeshkian et al., 2016). Also, Shiga toxins cause bloody diarrheal symptoms in patients (Bryan, Youngster, & McAdam, 2015). *Stx* genes are the molecular markers for detection and identification of Shiga toxin‐producing pathogens in food and clinical samples. However, detection of these genes is not solely adequate to confirm that the isolates are pathogenic (Parsons, Zelyas, Berenger, & Chui, 2016). Considerable prevalence of *E. coli* serotype O157: H7 and *Sh. dysenteriae* type 1 as the most known Shiga toxin‐producing pathogens were recently reported in many food items including ground meat, raw milk, vegetable salads, and fast food products by several researchers (Amani, Ahmadpour, Fooladi, & Nazarian, 2015; Bai et al., 2015; Dong et al., 2017).

Because of the higher prevalence of foodborne illnesses in developing countries, rapid, precise, specific, sensitive and inexpensive methods are appreciated to be employed and developed for detection and identification of foodborne pathogens. Several researchers have developed rapid detection methods based on antibody–antigen reaction, nucleic acid sequence difference, and bacterial metabolites in the recent decades. All of these assays were highly specific and sensitive in comparison with the gold standard ones including culture‐based and serological methods for detection of foodborne pathogens (Law, Ab Mutalib, Chan, & Lee, 2015). Also, different polymerase chain reaction (PCR)‐based techniques as rapid methods have been used in several studies for identification of foodborne pathogens. In PCR, specific sequence of a gene present in the genome of the target pathogen is detected by an enzymatical amplification procedure following characterization of the reaction products (amplicons) with different assays (Watson, 2012). In the simple PCR assay, amplicons are characterized by agarose gel electrophoresis and DNA standard marker (DNA ladder) to determine the length of the amplicons, consequently, identify the specific amplicons (Rahman, Uddin, Sultana, Moue, & Setu, 2013). PCR amplicon characterization methods including gel electrophoresis and melting temperature or curve assays have been developed and employed with higher sensitivity, specificity, and accuracy by researchers for identification of the target sequence in different PCR‐based techniques (Yanagihara et al., 2010).

By the emergence of the third‐generation intercalating dyes such as LCGreen® and EvaGreen®, PCR high resolution melting curve analysis (HRMA) method was developed to precisely characterize and identify the amplicons as a PCR‐based technique. This method was developed and implemented for the first time by Dr. Carl T. Wittwer in the year 2003 (Wittwer, Reed, Gundry, Vandersteen, & Pryor, 2003). Intercalating dyes with fluorescence emission bind to and saturate the double strands DNA (dsDNA). While the temperature is rising, dsDNA dissociates to the single‐strand DNA (ssDNA); then, the intercalating dye releases from the ssDNA structure. Releasing of the dye leads to emission of fluorescence from the reaction tube which is recorded by the machine at each temperature to create the melting curve of amplicon. In HRMA, third‐generation saturating intercalating dye, raising temperature, and recording fluorescence with higher resolution and precision are employed to construct precise melting curves; then, they are analyzed by statistical procedures (Wittwer, 2009). Small variations including deletion, insertion, and single nucleotide polymorphism (SNP) in DNA sequences can be detected by this assay (Farrar & Wittwer, 2017). High resolution melting curve analysis of PCR products can identify differences among the almost similar sequences with small variations as exists between the *stx1A* genes present in the genome of *Escherichia coli* O157: H7 and *Shigella dysenteriae* type 1. The goal of this study was to develop HRMA PCR assay for differentiation and detection of *stx1A* gene to identify *E. coli* O157: H7 and *Sh. dysenteriae* type 1 isolated from food samples and determination of prevalence of these pathogens by this method.

## 2 MATERIALS AND METHODS

### 2.1 Bacterial strains and food samples collection

*Escherichia coli* serotype O157: H7 ATCC 43,895 and *Sh. dysenteriae* type 1 ATCC 13,313 were employed as positive control strains. Negative control strains used at the present study for specificity and sensitivity evaluations of the assay included *Klebsiella pneumoniae* ATCC 13,883, *Proteus mirabilis* ATCC 35,659, *Enterobacter cloacae* ATCC 13,047, *Yersinia enterocolitica* ATCC 9,610, *Citrobacter rodentium* ATCC 51,459, *Salmonella* enterica ATCC 35,664, and non‐O157 Shiga toxin‐producing *E*. *coli* strains (non‐O157 STEC including *E*. *coli* O111 ATCC BAA‐2440 and *E*. *coli* O103 ATCC MP‐9 which can be detected by the primers designed in this study). Lyophilized strains were activated by inoculation in Luria‐Bertani Broth medium (LB, Promedia, Spain) and incubation at 37°C for 24 hr. One hundred thirty‐five food samples including ground meat (200 gr), milk (1 L), and vegetable salad (200 gr)(45 samples each food item) were collected from retail stores located in different regions of Qazvin city, Iran, from Jun to September 2019, for detection of *E. coli* serotype O157: H7 and *Sh. dysentariae* type 1; also, evaluation of PCR assay with HRM curve analysis. All samples were immediately transported to the Food Microbiology Laboratory of Veterinary Medicine Faculty, University of Tehran under cold condition (<4°C) and stored in a refrigerator until the microbial isolation (Hoseinpour, Foroughi, Nomanpour, & Nasab, 2017).

### 2.2 Culture‐based and the gold standard methods for detection and identification of the isolates

**Figure 1:**
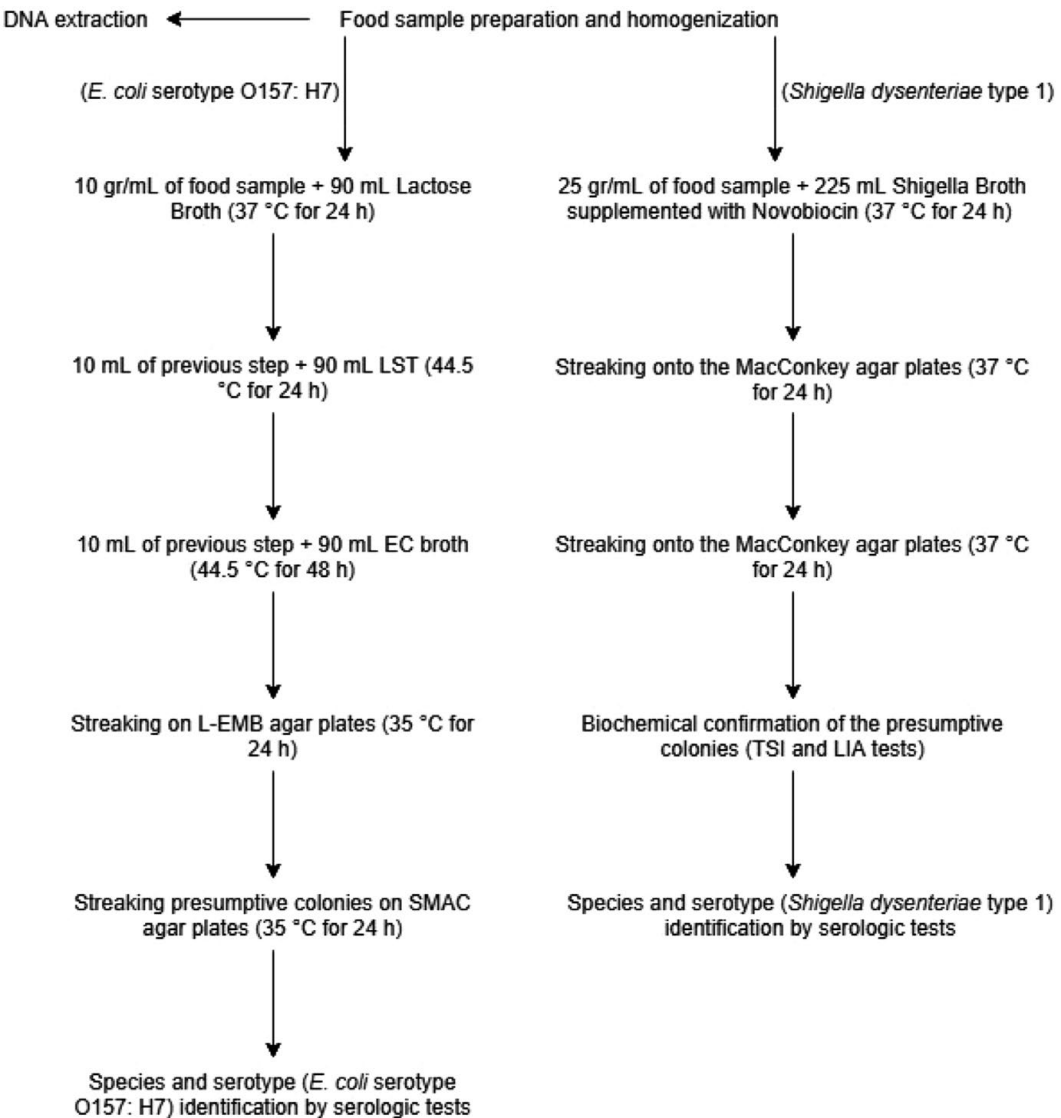
Flowchart of golden standard and conventional methods for detection and identification of *Escherichia coli* serotype O157: H7 and *Shigella dysenteriae* type 1 in food samples

For isolation and identification of *E. coli* serotype O157: H7 and *Sh. dysentariae* type 1 in food samples, culture‐based and serological identification methods described by *Microbiological Examination Methods of Food and water protocols* were used as gold standard procedures (Da Silva et al., 2018). For sample preparation, 25 gr for ground meat and vegetable salad, and 25 ml of milk samples were blended with 225 ml of buffered peptone water broth medium (Promedia, Spain) then homogenized by Stomacher BagMixer Lab blender (InterScience, France) for 1 min. Homogenized samples were subjected to isolation procedure (Golden standard methods) and DNA extraction (Figure 1). After isolation, presumptive colonies were subjected to serologic tests using Difco Antisera Kit (BD‐Difco Co. USA) for confirmation of the strains according to the kit manufacturer instructions.

### 2.3 DNA extraction

The total nucleic acid was extracted from food samples employing SinaColon commercial total DNA extraction kit (SinaClon Co., Iran). The DNA extraction procedure was performed according to kit manufacturer's instructions. The concentration of the extracted DNA was adjusted to 50 µg/ml using NanoDrop spectrophotometer (Thermo Fisher Science Co., USA) at 260 nm. The purity of the extracted DNA samples (260/280 ratio) was between 1.82 and 1.96.

### 2.4 Primer design

**Figure 2:**
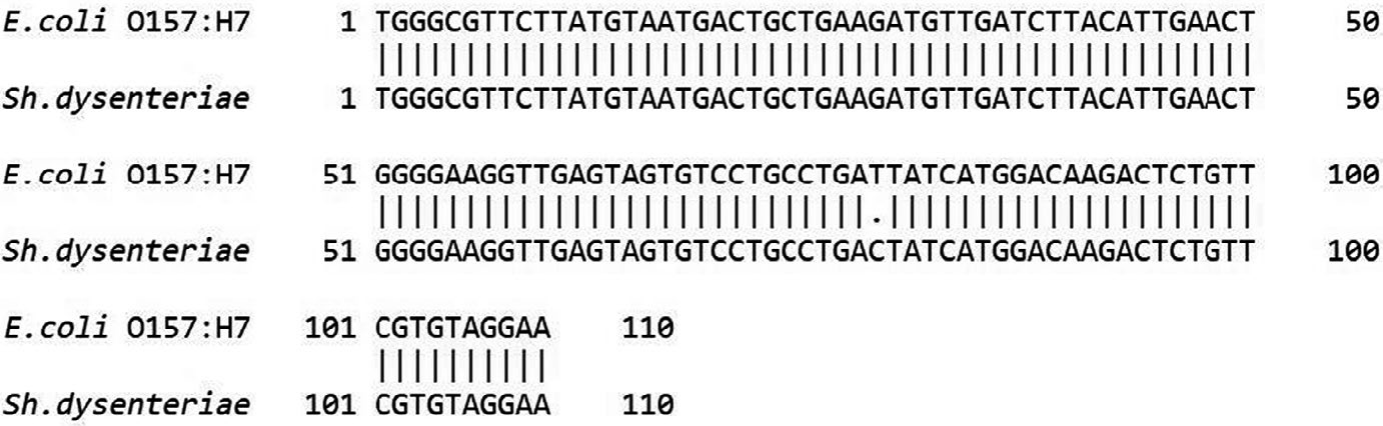
Pairwise sequence alignment of *Stx1A* gene for *Escherichia coli* serotype O157: H7 and *Shigella dysenteriae* type 1

Specific primer pairs for identification of *E. coli* serotype O157: H7 and *Sh. dysenteriae* type 1 were designed based on detection of *stx1A* gene‐encoded Shiga toxin released by these pathogens using IDT PrimerQuest online tool to design specific primers for qPCR intercalating dye reaction (https://eu.idtdna.com/Primerquest/Home/Index) (IDT, USA). Before designing specific primers, pairwise sequence alignment using EMBOSS Needle pairwise sequence alignment online tool (https://www.ebi.ac.uk/Tools/psa/emboss_needle/) was implemented to determine the different sequence region between genes of two pathogens. As it can be seen in Figure 2 and according to the result of the alignment, one nucleotide is the difference between *stx1A* gene sequences between these pathogens. Consequently, this region sequence was employed for specific primer design to run PCR reaction and HRM analysis. The specific primers were designed so as keeping the melting temperature (T_m_) of the amplicons between 80°C and 90°C to differentiate aligned genes among *E. coli* serotype O157:H7 and *Sh. dysenteria*e type 1. Table 1 shows the designed specific primers employed at the present study. The T_m_ of the amplicon was measured using IDT OligoAnalyzer online tool (https://eu.idtdna.com/calc/analyzer). Also, specificity of the designed primers for detection of *stx1A* gene in *E. coli* serotype O157:H7 and *Sh. dysenteriae* type 1 was evaluated using NCBI pimerBLAST online tool (http://www.ncbi.nlm.nih.gov/tools/primer‐blast/). The designed primers were synthesized by SinaClon company (SinaClon, Iran).

**Table 1:** Primer sequences for detection of *Escherichia coli* O157: H7 and *Shigella dysenteriae* type 1 by high resolution melting curve analysis

Primer	GeneBank no.	Sequence	Amplicon size (bp)
Stx1AF	NC_028685.1	3`‐TGGGCGTTCTTATGTAATGACT−5`	110
Stx1AR		5`‐TTCCTACACGAACAGAGTCTTG−3`	

### 2.5 PCR assay and HRM curve analysis

High resolution melting PCR assay of DNA samples was performed in a total reaction volume of 20 µl containing 10 µl of premix Hot start Taq (TEMPase hot start master mix, Ampliqon, Denmark), 1 µl of each specific primers (200 nM), 2 µl of LCGreen® plus dye (Idaho Technologies Inc., USA), 0.5 µl BSA (10 mg/ml), 2 µl DNA template, and deionized nuclease‐free water for bringing the total reaction volume to 20 µl. Nuclease‐free deionized water was employed as the no template control in the assay. Corbett Rotor Gene 6,000 real‐time PCR thermocycler (QIAGEN, Australia) was used with the following thermocycling program: 95°C for 10 min, 35 cycles at 95°C for 15 s and 62°C for 35 s; then, high resolution melting curve was recorded from 65°C to 95°C with a melting rate of 0.2°C/s. All obtained data were analyzed by manufacturer`s software of Corbett Rotor Gene 6,000 machine ver. 1.7.87. The confidence level of 90% was considered for genotype group clustering of amplicon high resolution melting profiles by the software.

### 2.6 Sensitivity and specificity of the assay

For assessment of the HRMA assay for identification of *E. coli* serotype O157:H7 and *Sh. dysentariae* type 1 in food samples, sensitivity and specificity of the assay should be evaluated. Sensitivity and specificity characteristics of the employed method were evaluated by the following formulas: Se = TP/(TP + FN) and Sp = TN/(TN + FP), where Se is sensitivity, TP is true positive (Detected by the assay and gold standard method), FN is false negative (Not detected by the assay but identified by the gold standard), Sp is specificity, TN is true negative (Not detected by the assay and gold standard), and FP is false positive (Detected by the assay but not detected by the gold standard) (Xiao et al., 2014).

### 2.7 Statistical analysis

Fisher's exact and chi‐square tests were used for evaluation of significant differences (*p* < .05) between contamination rates of different food item group samples using SPSS software version 22.0.1 (Chicago, IL, USA). All statistical and experimental measurements were performed in triplicate.

## 3 RESULTS AND DISCUSSION

### 3.1 Identification of *stx1A* using HRMA method and assessment of the assay

**Figure 3:**
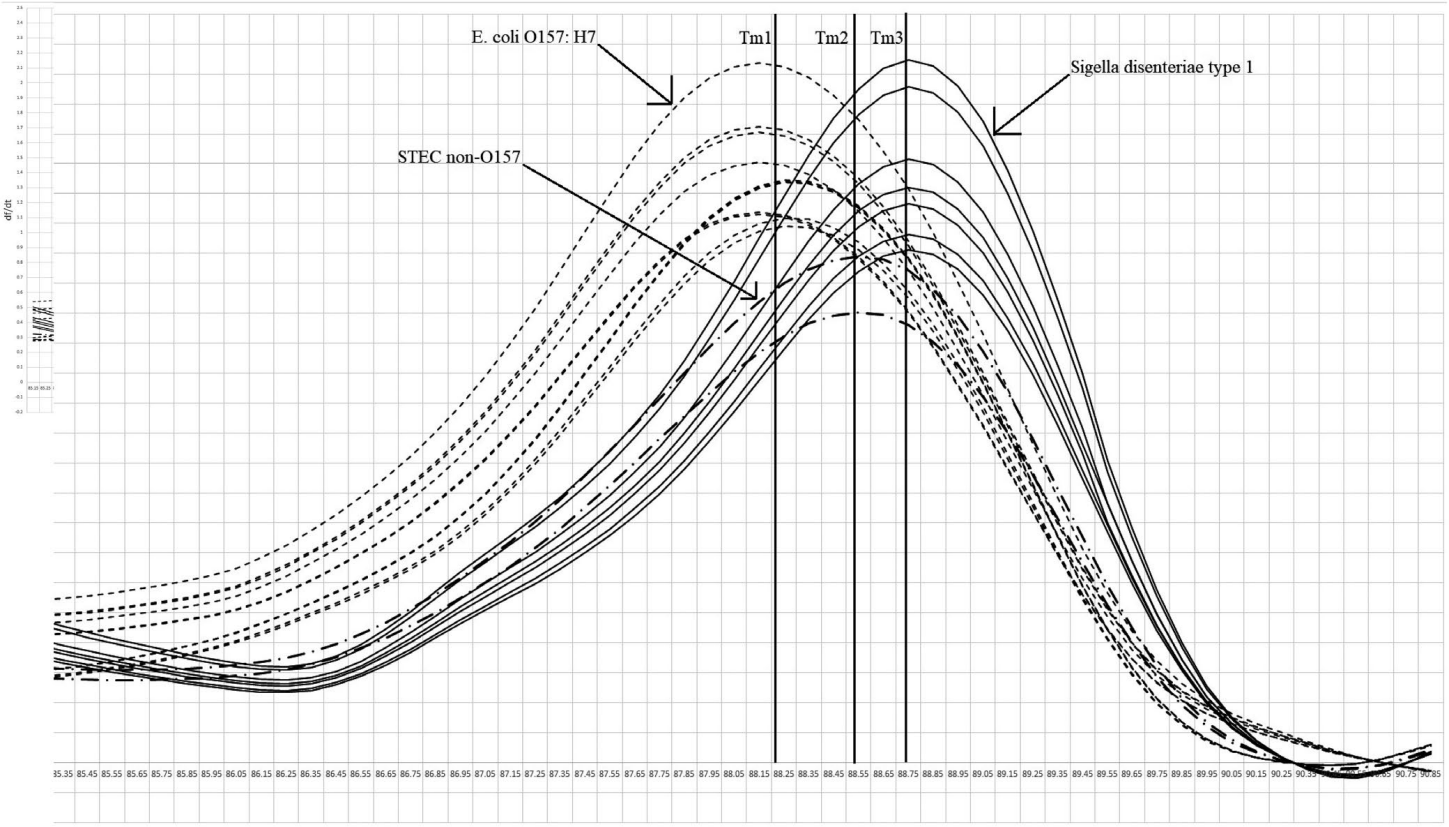
Melting curves and melting temperatures of the *stx1A* gene PCR product for detection of *Escherichia coli* serotype O157: H7, non‐O157 STEC, and *Shigella dysenteriae* type 1 (T_m1_, T_m2,_ and T_m3,_ respectively)

**Figure 4:**
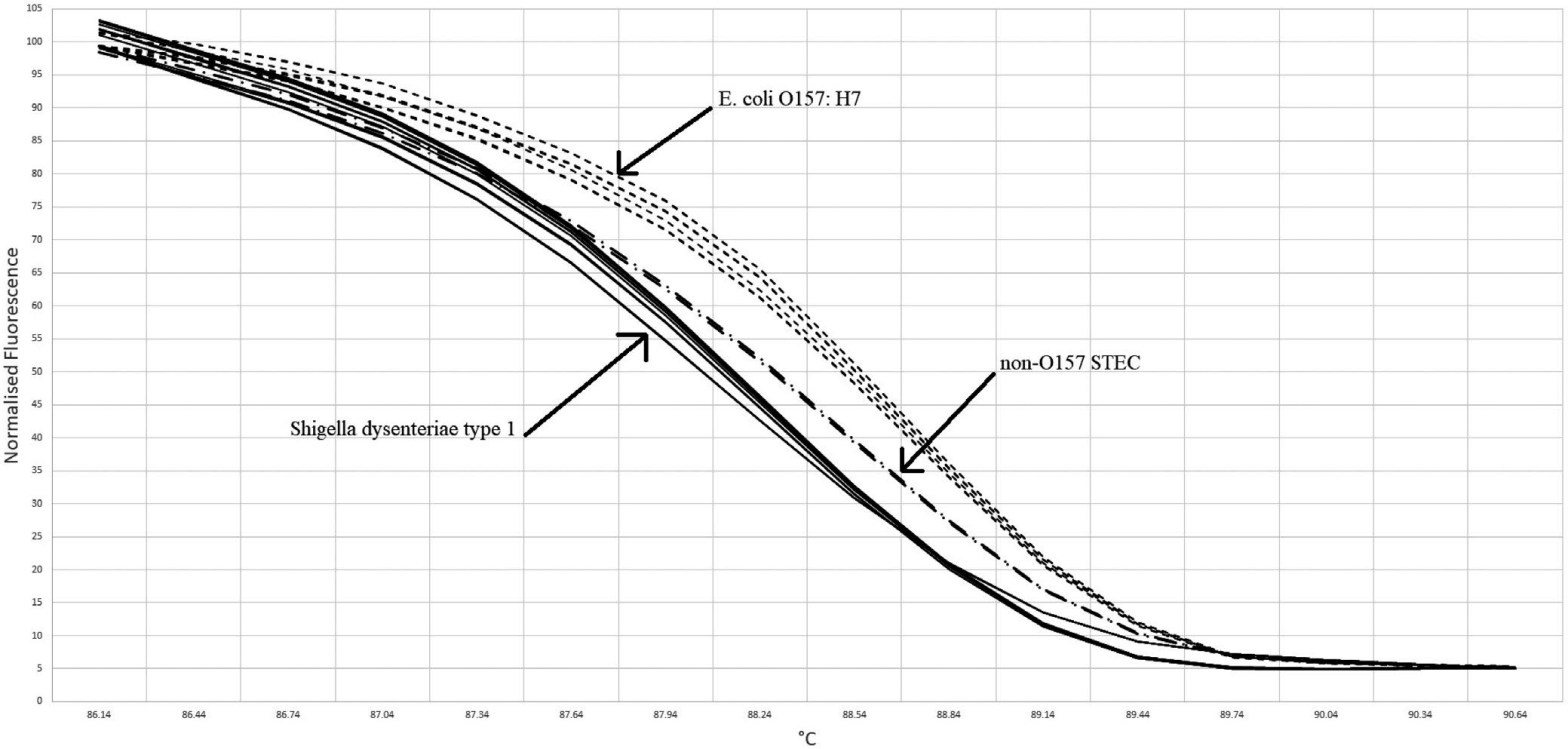
Normalized melting curves of the *stx1A* gene PCR product for detection of *Escherichia coli* serotype O157: H7, non‐O157 STEC, and *Shigella dysenteriae* type 1 isolates with confidence level >90%

*stx1A* gene for detection of *E. coli* serotype O157: H7 and *Sh. dysenteriae* type 1 strains was identified successfully using HRM curve analysis assay; also, it was employed for food samples as an accurate, specific and sensitive method for differentiating two pathogens. We identified three distinguishable melt curves to detect *stx1A* gene of both pathogens and non‐O157 STEC included in the study (Figure 3). Melting curves are categorized into *E. coli*, *Sh. Disenteriae,* and non‐O157 STEC groups. Reference strains were identified for each group. Our results revealed that difference in sequence of the *stx1A* gene (one nucleotide: T → C as shown in Table 1) presents in *E. coli* and *Sh. dysenteriae* strains significantly changes the shape and transition the T_m_ of the melting curves as provided in Figure 3. The melt curve analysis of the isolates showed that T_m_ of 10 and 6 curves of *E. coli* and *Sh. dysenteriae* type 1, respectively, were categorized to different groups without any non‐O157 STEC curve. Also, non‐O157 were differentiated from our isolates using HRMA in the study. We measured melting temperatures of the *stx1A* PCR product for isolated *E. coli* O157: H7, non‐O157 STEC, and *Sh. dysenteriae* 88.2 (T_m1_), 88.5 (T_m2_), and 88.7°C (T_m3_), respectively. Normalized and difference fluorescence curves show significant differences between the groups of melting curves with considering significant confidence levels in HRMA method. Normalized fluorescence curves of the melting curves (Figure 4) provided that three significant groups of melting curves reaching 100, 98.6, and 99.8% confidence levels for *E. coli,* non‐O157 *E. coli,* and *Sh. Dysenteriae,* respectively, were also differentiated with each other by HRMA method.

**Figure 5:**
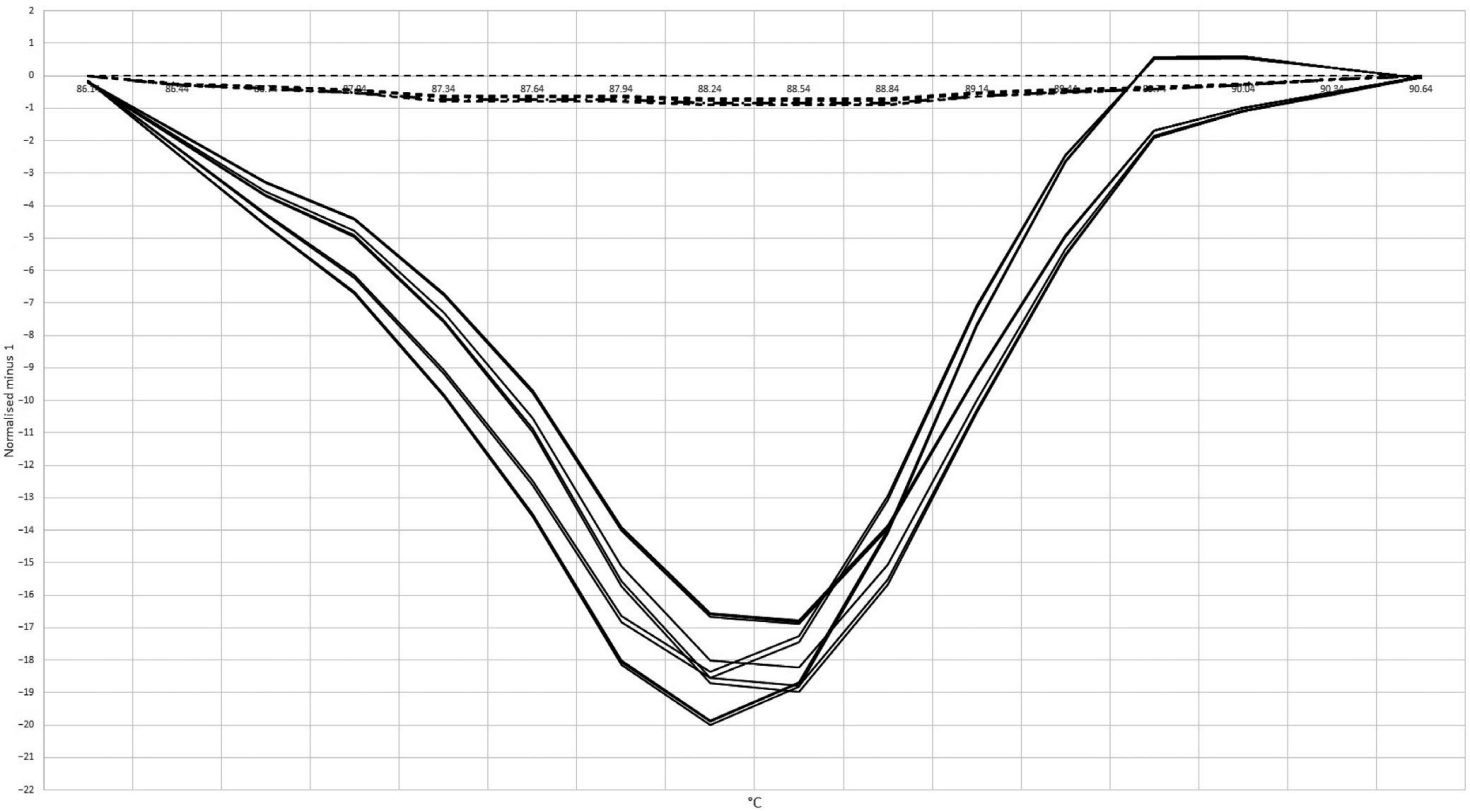
Difference melting plots of *stx1A* gene PCR product for detection of *Escherichia coli* serotype O157: H7 (dotted lines), non‐O157 STEC (dashed lines), and *Shigella dysenteriae* type 1 (solid lines) isolates

These results showed that this method can be employed confidentially to differentiate *stx1A* PCR products. Therefore, we detected *E. coli* serotype O157: H7 and *Sh. dysenteriae* type 1 strains in food samples successfully. Significant different categorization of the difference melting plots (Figure 5) also provided that we differentiate *stx1A* melting curves to detect these pathogens by HRMA successfully (Bezdicek et al., 2016). All strains identified by HRM assay from unknown samples were detected positive using gold standard method (No fluorescence signal was recorded in samples reported negative by the gold standard method) indicate that there are only true positive results. Also, the number of the true positive and negative samples analyzed with both methods was the same (Xiao et al., 2014). Consequently, specificity and sensitivity of the method were 100 and 100%, respectively, for differentiation of *stx1A* gene to detect *E. coli* serotype O157:H7, non‐O157 STEC, and *Sh. dysenteriae* type 1 strains in food samples.

*Shigella dysenteriae* type 1 and *E. coli* O157: H7 are Shiga toxin‐producing bacteria and the main causes of bacilli bloody diarrhea and HUS diseases in human. These pathogens usually transmitted from food to human as critical factors of foodborne outbreaks (Mellor et al., 2015). *Stx* genes encoding Shiga toxin proteins are presented in *Sh. dysenteriae* type 1, *E. coli* O157: H7, and other Shiga toxin‐producing bacteria (non‐O157 STEC). The sequence of this gene is more similar between *Sh. dysenteriae* type 1 and *E. coli* O157: H7 than other strains (Amani et al., 2015). Considering difference between *stx* gene (*stx1A*) sequence between these pathogens, some methods such as HRMA based on saturating intercalating dye and melting curve analysis of PCR product can be employed and developed as a rapid identification and detection assay. As shown at the present study, PCR coupled with HRMA, with 100% specificity and sensitivity, could be considered as an alternative assay for culture‐based method to precisely detect *Sh. dysenteriae* type 1 and *E. coli* O157: H7 in food samples. Rapid detection is a key factor of food microbial monitoring to track the outbreaks and contamination of imported and exported food items as quickly as possible (Lee, Runyon, Herrman, Phillips, & Hsieh, 2015). In comparison with gold standard method including isolation, identification, and serologic tests of presumptive isolates, HRMA assay is more inexpensive alternative method to detect pathogen in total DNA extracted from food sample (Iacumin et al., 2015). Amani et al. (2015) developed PCR‐ELISA assay for identification of *stx* genes in *E. coli* O157: H7 and *Sh. dysenteriae* strains isolated from clinical samples. They found PCR‐ELISA assay a sensitive, specific (100 and 100%, respectively) and rapid method for detection of Shiga toxin encoded gene for identification of these pathogens (Amani et al., 2015). However, this assay is more expensive than PCR‐HRMA method.

Despite the limited researches implemented on detection of *E. coli* O157: H7 and *Sh. dysenteriae* by identification of *stx* gene or other pathogen markers, there are several studies developed PCR‐HRMA assay for detection of virulence factors for rapid detection of pathogens in food and clinical samples. Forghani, Wei, and Oh (2016) employed HRMA as a rapid multiplex method for identification of *Bacillus cereus*, *Staphylococcus aureus,* and *Listeria monocytogenes* isolated from different food samples simultaneously. They found HRMA a rapid, sensitive, specific and economical assay to simultaneous identification of these pathogens in food samples (Forghani et al., 2016). Also, Hoseinpour et al. (2017) used PCR‐HRM for identification and differentiation of *cadF* gene to detect *Campylobacter* species in chicken samples. They reported this rapid identification assay specific and sensitive (Hoseinpour et al., 2017). There are many recent studies using HRMA specially to identify pathogens in clinical and food samples. They found this method highly sensitive and specific (100 and 95%, respectively). The precision of the different normalized melting curves makes HRMA an accurate method to differentiate marker genes between the different microbial genus and species with the same sequence (Druml & Cichna‐Markl, 2014). Also, we found HRMA in our study as a suitable method to differentiate *stx1A*, as a virulence factor marker gene, to identify *E. coli* O157: H7 and *Sh. dysenteriae* in food samples. Presence of these pathogens is enough for compromising the safety of the food samples (positive result of each pathogen in one gram or milliliter of the sample); consequently, only detection of these pathogens has the superiority over the quantitative methods. However, this method probably can be employed for detection and differentiation of *stx1A* gene to detect these pathogens in clinical specimen as a sensitive, specific, rapid and especially inexpensive method in comparison with the conventional and serologic gold standards.

### 3.2 Prevalence of *E. coli* serotype O157: H7 and *Sh. dysenteriae* in food samples identified by *stx1A* gene detection using HRMA assay

**Figure 6:**
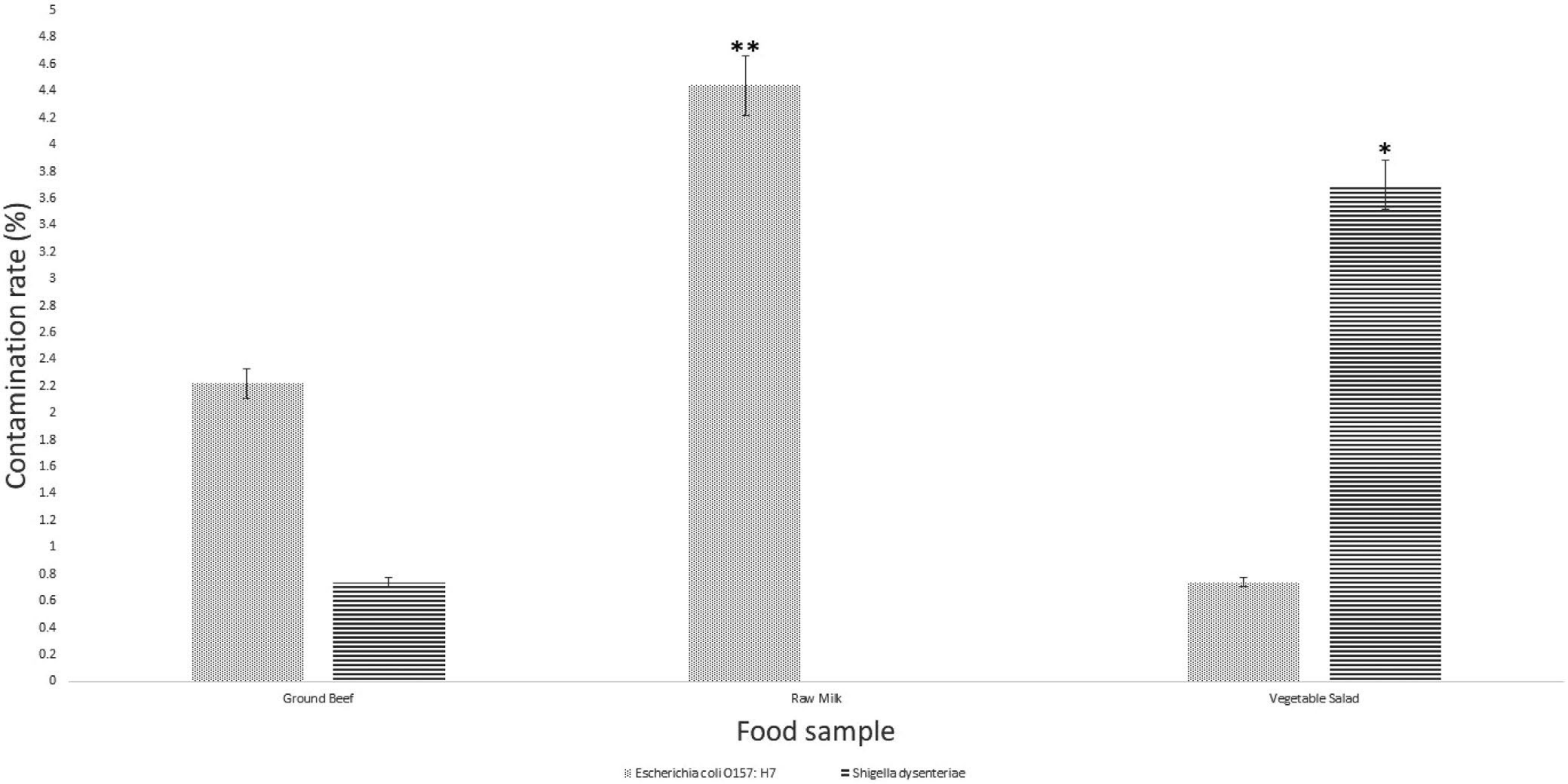
Prevalence and contamination rate of *Escherichia coli* serotype O157: H7 and *Shigella dysenteriae* type 1 in food samples detected by HRMA method

It is worthwhile noting that the prevalence of *E. coli* O157: H7 and *Sh. dysenteriae* type 1 in food samples is recently deceased but in developing countries the contamination rate of these pathogens is still remarkable (Law et al., 2015). The initial source of contamination with *E. coli* O157: H7 and *Shigella* spp. are intestinal system reminds of the cattle and human, respectively. *E. coli* O157: H7 previously reported in raw milk, vegetable salad and meat products (Currie et al., 2017). Many researchers also detected *Sh. dysenteriae* type 1 in vegetable salad and meat food items. There are not any studies reported *Sh. dysenteriae* in milk samples as it was not discovered in our study (Figure 6) (Weis et al., 2017). As provided in Figure 5, the most contamination rates of *E. coli* O157: H7 and *Sh. dysenteriae* type 1 detected by both PCR‐HRMA and the gold standard methods were significantly detected in raw milk and vegetable salad samples, respectively. Detection of *stx1A* gene as a virulence factor and pathogen gene marker was performed for detection of these pathogens. Between food items, identification of *Sh. dysenteriae* type 1 and *E. coli* O157: H7 in vegetable salads and meat products usually reported by other researchers; however, we observed at the present study. Shahin, Bouzari, Wang, and Yazdi (2019) detected high prevalence of *Shigella* spp. from vegetable samples; however, they did not find any *Sh. dysentariae* in any samples (Shahin et al., 2019). Contamination of these food products was occurred probably because of food handlers for *Sh. dysenteriae* and naturally contaminated raw materials for *E. coli O157:H7* (Weis et al., 2017). Detection of Shiga toxin encoded genes is very prominent in safety assessment of the food samples; however, the presence of this gene is not adequate to make disease. It is suggested to consider and identify other virulent factors encoded genes in foodborne pathogens to accurately evaluate the safety of food products (Amani et al., 2015). As a matter of fact, PCR‐HRMA assay can be employed for detection of virulence factor genes in identification of pathogens.

## 4 CONCLUSION

*Stx1A* gene in *E. coli* O157: H7 and *Sh. dysenteriae* type 1 has the genetic difference in one nucleotide applied for developing PCR‐HRMA assay for differentiation of this gene to detect these pathogens in food samples. At the present study, we found HRMA method specific and sensitive (100 and 100%, respectively) in comparison with the gold standard methods for differentiation of *stx1A* gene to detect Shiga toxin‐producing foodborne pathogens in food samples. Also, the highest contamination rates of confirmed *E. coli* O157: H7 and *Sh. dysenteriae* type 1 were detected among the raw milk and vegetable salad samples, respectively. Finally, PCR assay coupled with HRMA as a rapid, inexpensive, specific and sensitive method is suggested to be employed for detection of *E. coli* O157: H7 and *Sh. dysenteriae* type 1 by differentiation of *stx1A* gene in food samples.
